# Inflammatory myofibroblastic tumor of the pancreatic head – a case report of a 6 months old child and review of the literature

**DOI:** 10.2478/raon-2014-0017

**Published:** 2015-08-21

**Authors:** Ales Tomazic, Diana Gvardijancic, Joze Maucec, Matjaz Homan

**Affiliations:** 1 Department of Abdominal Surgery, University Medical Center Ljubljana, Ljubljana, Slovenia; 2 Department of Gastroenterology, Hepatology and Nutrition, University Children’s Hospital, Ljubljana, University Medical Center Ljubljana, Ljubljana, Slovenia

**Keywords:** child, inflammatory myofibroblastic tumor, duodenopancreatectomy

## Abstract

**Background:**

Inflammatory myofibroblastic tumors are rare in the pediatric population. Most common localizations were reported in the lungs. A localization in the pancreas needs differentiation from other tumors and chronic pancreatitis. Treatment is surgical resection, although there are reports of treatment with oral steroids and radiation therapy.

**Case report.:**

A 6-month-old child was treated due to a tumor in the head of the pancreas. On admission he was jaundiced with pruritus. US and MRI confirmed pancreatic tumor. Preoperative biopsy wasn’t conclusive regarding the nature of the tumor. Duodenopancreatectomy was performed. Postoperative course was uneventful. Histologic examination confirmed the diagnosis of inflammatory myofibroblastic tumor. On follow up, he remained with no evidence of recurrence.

**Conclusions:**

A literature review revealed 10 cases of pancreatic inflammatory myofibroblastic tumors in the pediatric age group. Our patient is the youngest reported. Despite major resection, there were no complications. However, management of this child might be possible with steroids, but conservative treatment might be insufficient, especially in aggressive forms of tumors.

## Introduction

Inflammatory myofibroblastic tumors (IMT’s) are rare solid lesions that occur primarily in visceral and soft tissue. Most frequently they occur in the first two decades of life. The most common localizations of IMTs have been reported in lung, mesentery and omentum.^1^ These lesions have also been termed inflammatory pseudotumors, fibroxanthomas, fibrous histiocytomas, postinflammatory tumors and plasma cell granulomas. There are few hypotheses of the etiological factors responsible for development of the IMT. IMT can develop as a consequence of an inflammatory reaction to an underlying low grade malignancy. *Human herpes viruses* 3 and 8, *Eikenella corrodens* and *Epstain Barr virus* have been also proposed as possible infectious triggers of the IMT.^2^,^3^,^4^ It is speculated that the disease is provoked by deregulation of cytokine production caused by infection.

Clinically and radiologically, an IMT can be confused with malignancy. A localization of IMT in the pancreas is very rare and needs differentiation from other tumors and chronic pancreatitis.

The macroscopic appearance of IMT is usually well-circumscribed or multinodular, white, firm mass. Histological, IMT is composed of spindle-shaped myofibroblasts or fibroblasts accompanied by a mixed inflammatory infiltrate of eosinophils, plasma cells, and lymphocytes.^1^,^5^ Treatment is usually in the form of surgical resection, although there are recent reports of treatment with oral steroids.^6^ Some authors report also palliative treatment with radiation therapy.^7^

We present a case of 6 months old male, who was referred to our department due to an IMT of the pancreatic head, which caused jaundice and pruritus. To the best of our knowledge, this is the youngest child with this type of tumor published so far in the literature.

## Case report

A 6-months old boy was transferred to our hospital with a 4-days history of jaundice and pruritus. On examination he was jaundiced with no organomegaly. There was a rash on the trunk and extremities. Liver function tests revealed a direct bilirubin level of 109 μmol/L (normal below 17), alkaline phosphatas level of 11.62 μkat/L (normal up to 1.74) and γGT level of 3.23 μkat/L (normal up to 0.63).

**FIGURE 1.**
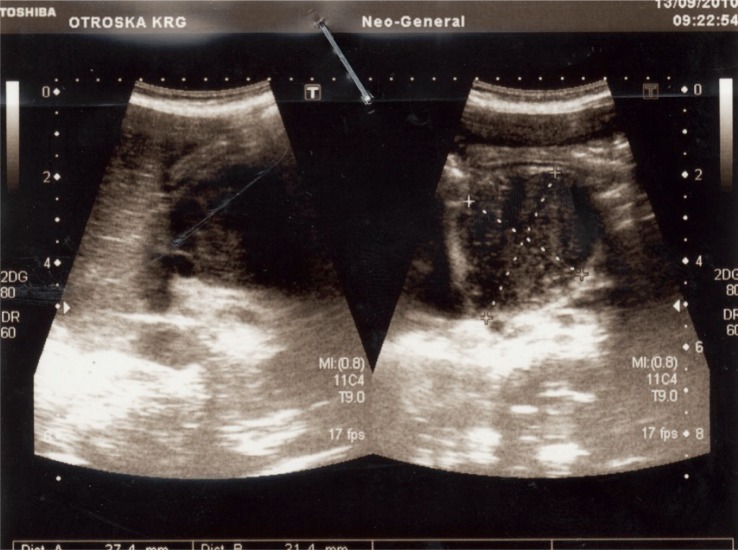
Ultrasound has identified 40 mm large mass in the region of the head of the pancreas.

**FIGURE 2.**
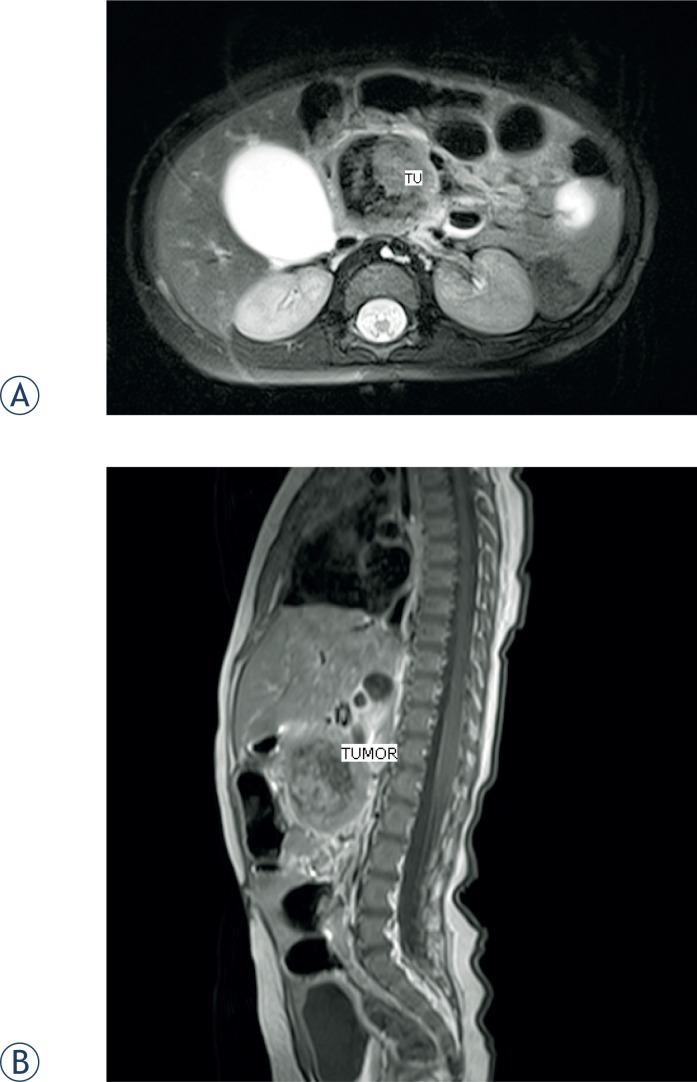
MRI confirmed well circumscribed tumor mass, with a diameter of 37 mm. The origin of the mass was in the head of the pancreas and in the uncinate process. There was no infiltration of the surrounding tissue. Tumor impressed the caval vein and pushed the superior mesenteric artery and vein ventraly and lateraly. **(A)** Coronal plane. **(B)** Sagital plane.

An ultrasound scan (US) of his abdomen identified a 40 mm large mass in the head of the pancreas. The common bile duct was dilated, the gall-bladder was extremely enlarged, but there was no dilatation of intrahepatic bile ducts and pancreatic duct (Figure 1). The results of US-guided fine needle aspiration biopsy wasn’t conclusive regarding the nature of the tumor. MRI confirmed well circumscribed tumor mass, with a diameter of 37 mm. The tumor originated from the head of the pancreas and uncinate process. 3D reconstruction showed no infiltration in the surrounding tissue, including major vessels (Figure 2). However, there was evidence, that the tumor impressed on the caval vein and pushed the superior mesenteric artery and vein ventrally and laterally.

**FIGURE 3.**
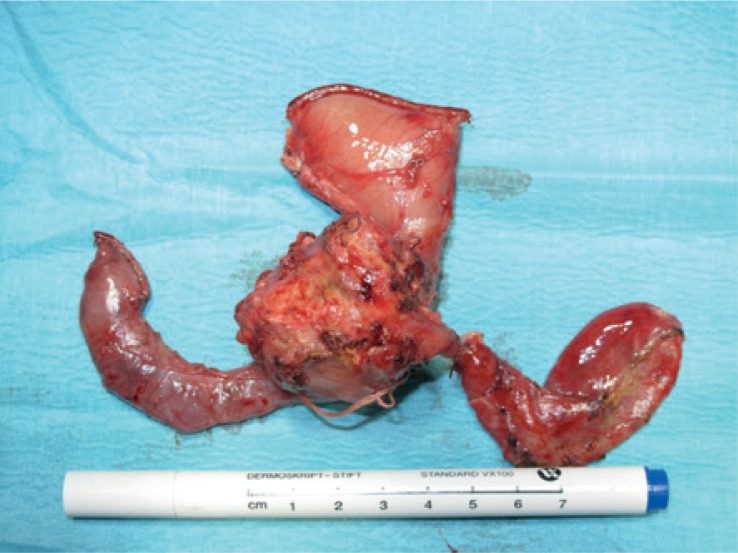
Surgical specimen of the duodenopancreatectomy. Left is duodenum, right gallbladder, up stomach and in the middle head of the pancreas with tumor.

Whipple’s procedure was performed due to biliary obstruction and possible malignancy (Figure 3).

**FIGURE 4.**
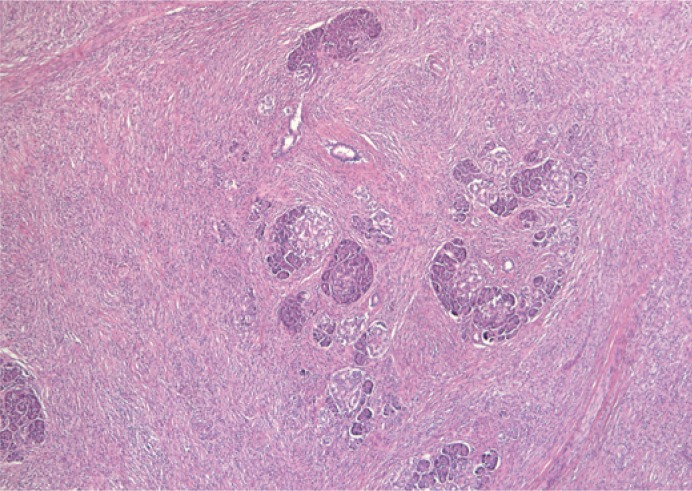
Spindle cell proliferation in the pacreatic head, growing in between and into the pacreatic acini.

**FIGURE 5.**
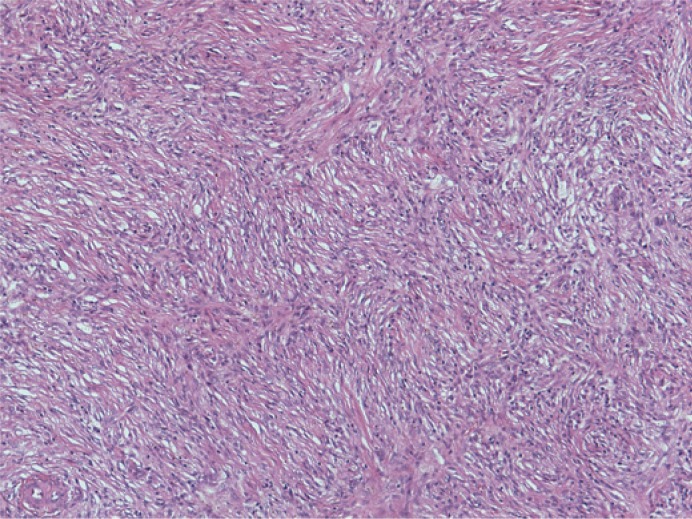
Bland spindle cell proliferation with vague storiform growth pattern.

**FIGURE 6.**
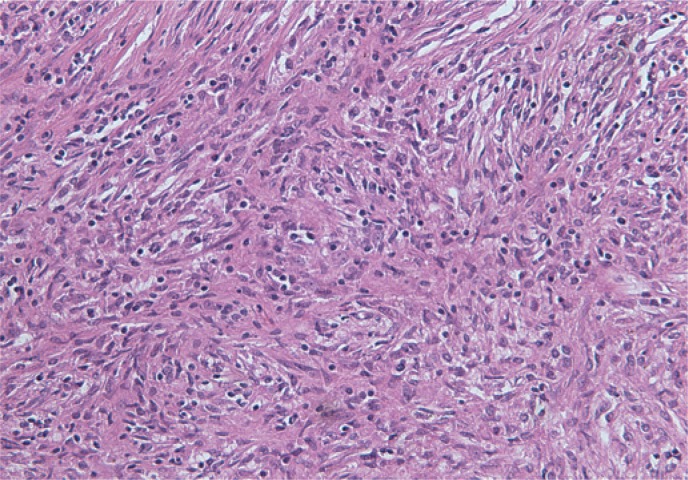
An admixture of spindled and more epithelioid lesional cells admixed with inflammatory cell infiltrate.

**FIGURE 7.**
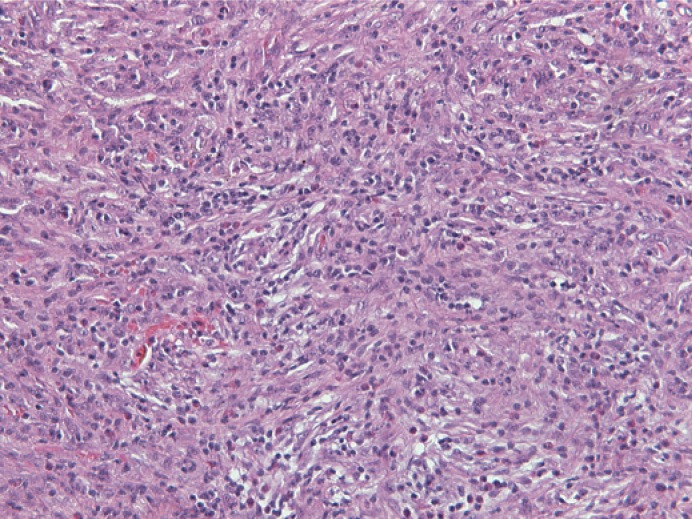
Prominent inflammatory cell infiltrate was present in several areas within the lesional cell proliferation.

**FIGURE 8.**
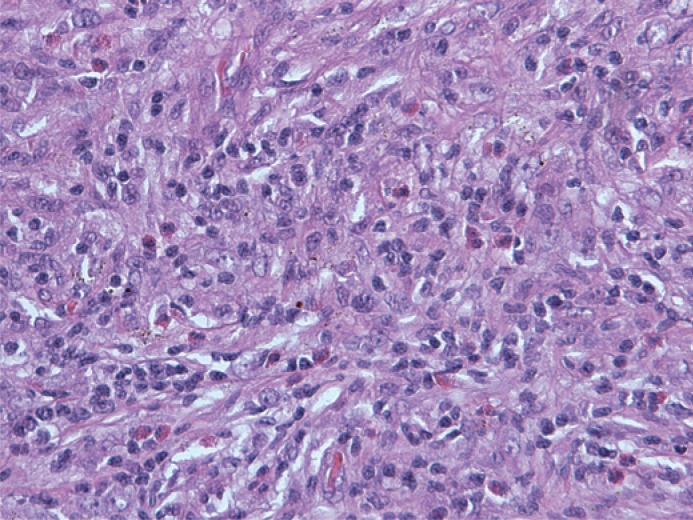
Note numerous eosinophilic granulocytes within the inflammatory cell component.

Histological examination revealed an infiltrative growth in the pancreatic head, mainly surrounding and destroying pancreatic acini (Figure 4), but also encroaching on papila of Vater and duodenal wall. The lesion was composed of bland spindle cells forming a storiform (Figure 5) and vague fascicular growth pattern, admixed with areas displaying more epithelioid morphology (Figure 6) and variably prominent inflammatory cell infiltrate (Figure 7), composed of lymphocytes, plasma cells and eosinophilic granulocytes (Figure 8).

By immunohistochemistry, the lesional cells were smooth muscle actin positive proliferation of bland spindle cells forming a storiform and vague fascicular growth pattern, admixed with areas displaying more epithelioid morphology and variably prominent inflammatory cell infiltrate, composed of lymphocytes, plasma cells and eosinophilic granulocytes, while stainings for cytokeratins, S100, desmin, H-caldesmon and ALK were negative, confirming myofibroblastic differentiation of the lesional cells. Although the histological and immunohistochemical features were suggestive of inflammatory myofibroblastic tumour, an unusual form of chronic pancreatitis could not be reliably excluded.

The postoperative course was uneventful. The boy was discharged on the 14th postoperative day. Over the next 3.5 years of follow up, he remains well and with no clinical or radiological evidence of recurrence.

## Discussion and review of literature

Pancreatic tumors are rare in childhood, accounting for only 0.2% of childhood malignancies.^8^ Inflammatory myofibroblastic tumors are usually benign solid lesions of unclear etiology, commonly found in the lungs. The term inflammatory myofibroblastic tumor, commonly referred to as inflammatory pseudotumor in the previous literature, was initially proposed in 1990 in the study of inflammatory lesions of the pulmonary system.^9^ The majority of the cases that were reported in the literature as »inflammatory pseudotumor« of the pancreas, would probably now be classified as autoimmune pancreatitis and in rare cases they represent true »inflammatory myofibroblastic tumors«.^10^,^11^ Another nosological problem with inflammatory myofibroblastic tumor is differentiation from inflammatory fibrosarcoma, which was first reported as an invasive tumor with greater atypia of constituent fibroblasts or myofibroblasts than seen in inflammatory myofibroblastic tumor.^12^ According to Coffin, inflammatory myofibroblastic tumors are characterized with local invasion, vascular invasion and multifocal onset.^13^ Invasion of retroperitoneal connective tissue, duodenal wall and Vater’s papila was also seen in our case. This indicated, that the lesion was neoplastic. However in inflammatory fibrosarcoma, more aggressive behaviour is seen, including higher incidence of recurrence and death.^12^ Inflammatory myofibroblastic tumor and inflammatory fibrosarcoma have been speculated to be two lesions occupying the same spectrum, with reported cases of inflammatory myofibroblastic tumors probably including some low-grade examples of inflammatory fibrosarcoma.^14^

Histologically, inflammatory myofibroblastic tumors are characterized by irregular proliferation of myofibroblasts intermixed with inflammatory cells, mainly lymphocytes and plasmacytes. They are subcategorized into fibrohystiocytic type, plasma cell granuloma type, largely sclerosed or fibrosed type, hypocellular fibrous type and myxoid/vascular type.^15^ Discovery of cytogenetic aberrations in inflammatory myofibroblastic tumors and the recognition of ALK gene rearrangements solidified the concept of inflammatory myofibroblastic tumor as a neoplastic lesion. It most frequently occurs in the lung or the mesenterium of children or young adults and rarely metastasizes (<5%).^16^ The liver is also relatively frequently involved^17^, but other sites such as the stomach^18^, spleen^19^, bladder^20^, kidney^21^, maxillary sinuses^22^, heart^23^, parapharyngeal space^24^, retrorectal space^25^ and peripheral nerve^26^ have also been recorded.

Only 28 cases of pancreatic inflammatory myofibroblastic tumors have been reported so far, 60% being located in the pancreatic head.^7^ Inflammatory fibroblastic tumor equally affects males and females. The age distribution resembled that of in pulmonary system ranging 2.5 to 70 years.^27^ A literature review revealed 10 documented cases of pancreatic inflammatory myofibroblastic tumor in the pediatric age group (Table 1).

Compared to this data, our patient is the youngest child with inflammatory myofibroblastic tumor of the pancreas reported in the literature. The main features at presentation were pruritus, jaundice, abdominal mass, lethargy, vomiting, fever and anemia.^6^ Curative resection is treatment of choice for inflammatory myofibroblastic tumors. Whipple’s procedure or distal pancreatectomy is performed, according to the site of the tumor. The prognosis of inflammatory myofibroblastic tumors is generally good, with rare incidence of malignant transformation.^28^ However, a significant recurrence rate of 25% was reported.^29^ It was suggested that the presence of atypia, ganglion-like cells and p53 expression may suggest more aggressive behaviour.^30^,^31^ These lesions may be indistinguishable from inflammatory fibrosarcoma due to a high degree of clinical and morphological overlap.^28^

Besides surgical resection, alternative therapeutic regimens are still lacking. While systemic immunosuppresive treatment with steroids, chemotherapy and radiation therapy have been reported for unresected or recurrent cases of extrapancreatic inflammatory myofibroblastic tumors.^28^,^32^,^33^

In the literature there are only two reported cases of pancreatic inflammatory myofibroblastic tumors that were not treated with resection.^6^,^7^ The first reported case was a child treated with high dose steroids. The mass gradually resolved and the patient remains disease free 6 years after treatment.^6^ The second case was an adult treated with palliative radiation and corticoid therapy because of unresectable mass in the head of the pancreas.^7^ In this case, long term results were not published.

Chronic pancreatitis could not be completely excluded according to histological examination. Anyway, specific causative factor for chronic pancreatitis was not identified. In addition, pediatric patients present with chronic pancreatitis much later (average age 6 ± 4 years) than it developed in our patient (6 months).^34^ Therefore, chronic pancreatitis was very unlikely the reason for pancreatic head mass in our patient.

## Conclusions

We report a case of pancreatic inflammatory myofibroblastic tumors in a six month old male child treated with surgical resection. This is the first case report of an infant with IMT. In addition, the tumor is rarely described in the pancreas. Despite major surgery no complications evolved in long term follow up.

## Figures and Tables

**TABLE 1.** Reported cases of pediatric pancreatic inflammatory myofibroblastic tumors in the literature

**Age**	**Sex**	**Location**	**Presentation**	**Treatment**	**Reference**
10	F	body	Abdominal mass	Distal pancreatectomy	Abrebanel et al. 1984
2.5	F	body	Anemia, fever, abdominal mass	Distal pancreatectomy	Scott et al. 1988
5	F	head	vomiting	Whipple	Stringer et al. 1992
8	F	head	Jaundice, anemia, weight loss	Whipple	Uzoaru et al. 1993
8	F	body	Abdominl mass	Distal pancreatectomy	Shankar eta al. 1998
11	F	head	Jaundice, pruritus, anorexia, pain	Whipple	McLain et al. 2000
4	F	head	Malaise, lethargy	Whipple	Slavotinek et al. 2000
11	M	body	Lethargy, anemia, abdominal mass	Distal pancreatectomy	Morris-Stiff et al. 1998
13	F	head	Jaundice, vomiting, weight loss	Whipple	Dagash eta l. 2009
10	M	head	Jaundice, pain	Steroids	Dagash eta l. 2009
